# Perinatal Factors and Regional Brain Volume Abnormalities at Term in a Cohort of Extremely Low Birth Weight Infants

**DOI:** 10.1371/journal.pone.0062804

**Published:** 2013-05-09

**Authors:** Nehal A. Parikh, Robert E. Lasky, Kathleen A. Kennedy, Georgia McDavid, Jon E. Tyson

**Affiliations:** 1 Department of Pediatrics, University of Texas Medical School at Houston and Children's Memorial Hermann Hospital, Houston, Texas, United States of America; 2 Center for Perinatal Research, The Research Institute at Nationwide Children’s Hospital and The Ohio State University College of Medicine, Columbus, Ohio, United States of America; 3 Center for Clinical Research and Evidence-Based Medicine, University of Texas Medical School at Houston, Houston, Texas, United States of America; University of Cambridge, United Kingdom

## Abstract

Our objective was to investigate diverse clinical antecedents of total and regional brain volume abnormalities and white matter hyperintensity volume on term MRI in extremely low birth weight (birth weight ≤1000 g) survivors. A consecutive cohort of extremely low birth weight infants who survived to 38 weeks postmenstrual age (n = 122) and a control group of 16 healthy term newborns underwent brain MRI at term-equivalent age. Brain volumes were measured using semi-automated and manual segmentation methods. Using multivariable linear regression, clinical antecedents were correlated with volumes of total brain tissue, white matter hyperintensities, and regional tissues/structures, adjusted for age at MRI, total cranial volume, and total tissue volume. Regional brain volumes were markedly reduced in extremely low birth weight infants as compared to term newborns (relative difference range: −11.0%, −35.9%). Significant adverse clinical associations for total brain tissue volume included: small for gestational age, seizures, caffeine therapy/apnea of prematurity, duration of parenteral nutrition, pulmonary hemorrhage, and white matter injury (*p*<0.01 for each; relative difference range: −1.4% to −15.0%). Surgery for retinopathy of prematurity and surgery for necrotizing enterocolitis or spontaneous intestinal perforation were significantly associated with increasing volume of white matter hyperintensities. Regional brain volumes are sensitive to multiple perinatal factors and neonatal morbidities or interventions. Brain growth measurements in extremely low birth weight infants can advance our understanding of perinatal brain injury and development.

## Introduction

While survival rates for extremely low birth weight infants (ELBW; ≤1000 g) over the past two decades have improved dramatically, rates of neurodevelopmental impairments (NDI) remain alarmingly high [1], [2]. Indeed, the prevalence of impairments may be increasing [3], [4]. Regional brain volume abnormalities and white matter signal abnormalities on T2-weighted MRI scans, known as diffuse excessive high signal intensity (DEHSI), are the most common findings in ELBW infants on MRI at term-equivalent age [5]–[8]. In preliminary investigations, such abnormalities have been associated with cerebral palsy, memory deficits, and intellectual impairments [6], [9]–[13]. However, the neurodevelopmental significance of DEHSI remains controversial [12]–[15]. Development of more objective quantitative measures of DEHSI and regional volumes may reduce measurement error and resolve this debate [16].

Advanced MRI techniques such as volumetric and diffusion tensor MRI provide objective, quantitative, and sensitive measures of perinatal brain injury and development [5], [9], [17]–[19]. Preterm infants exhibit both global and regional brain volume abnormalities at term that persists into young adulthood [5]–[7], [18], [20], [21]. Yet, antecedents of regional brain volume measurements remain largely unknown [5], [7], [22]–[25]. Discovery of new modifiable risk factors may facilitate brain injury prevention and/or targeted treatments. Our aims were to determine the extent of regional brain volume abnormalities and white matter hyperintesity volume at term-equivalent age in ELBW infants as compared to healthy term controls and to examine perinatal antecedent factors associated with such abnormalities in a prospective cohort of ELBW infants.

## Patients and Methods

### Ethics Statement

The Institutional Review Board of the University of Texas Houston Medical School and Children’s Memorial Hermann Hospital approved the study. Written informed consent was obtained from parents of all term infants.

### Subjects

Of the 202 ELBW infants born at or transferred (within 14 days of birth) to Children’s Memorial Hermann Hospital NICU between August 2005 and January 2007, 140 ELBW infants survived to 38 weeks postmenstrual age (PMA). Six infants with congenital anomalies were excluded. All 134 eligible ELBW infants were enrolled in this cohort. A control group of 16 healthy 1- to 4-day old full-term newborns (37 to 41 weeks appropriate for gestational age) was recruited from our well-baby nursery between July 2008 and January 2010. The exclusion criteria for this group were: any congenital or chromosomal anomaly, multiple gestation pregnancy, maternal medical or pregnancy conditions, pre-delivery hospital admission, intrauterine exposures (drugs of abuse, cigarettes, alcohol, medications with known/suspected CNS effects [steroids]), high forceps or vacuum delivery, perinatal distress, NICU admission, abnormal neurologic exam or suspected/known infection at birth.

### Data Collection and Definitions

Trained research personnel collected a detailed list of maternal characteristics and pregnancy/delivery data and infant data from birth to discharge MRI. Definitions for maternal and infant characteristics were provided in a manual of operations that was originally developed by the NICHD Neonatal Research Network [26] and augmented for this study. Gestational age in completed weeks was defined using the best obstetrical estimate based on the last menstrual period, early ultrasonographic examination, or when these two were infrequently unavailable, by a Ballard examination by the pediatrician. Cranial ultrasound was routinely obtained at 10 to 14 days after birth. A summary list of antecedent factors and their definitions examined in our study are listed in Table S1.

### MRI Measurements

Brain MRI scans were routinely performed on all ELBW infants at 38 weeks PMA or prior to discharge for infants discharged before 38 weeks. A skilled transport nurse accompanied all infants to the MRI suite. After the infant was fed, silicone earplugs were placed (Instaputty, E.A.R. Inc, Boulder, CO), and the infant was swaddled with a blanket and/or vacuum immobilization device (MedVac, CFI Medical Solutions, Fenton, MI) to promote natural sleep and avoid sedation. MRI was performed on a 1.5T GE-LX scanner. Axial PD/T2w scans were used for volumetry with TE 15/175 ms; TR 10000 ms; FOV 18×18 cm; matrix 512×512; voxel dimensions: 0.36H×0.36W×1.98D mm. For the full-term cohort, MRI was performed on a 3.0T Philips scanner using similar sequence parameters: TE 9/175 ms; TR 10000 ms; FOV 18×18 cm; matrix 256×256; voxel dimensions: 0.70H×0.70W×2.00D mm. Forty-four slices covered the entire cerebrum and the brain stem.

### MRI Post-processing

**Figure 1:**
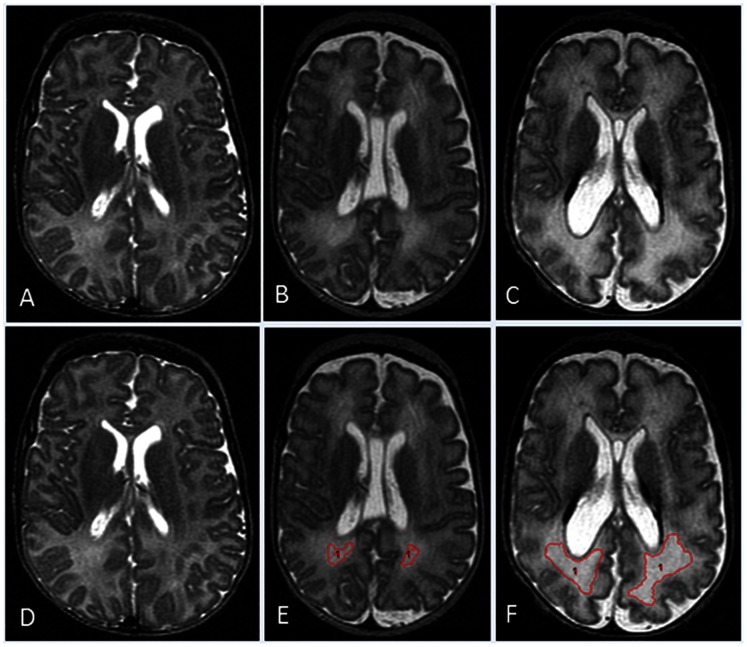
Different grades of WMH with semi-automated segmentation results demonstrated on mid-axial T2-weighted images from three different subjects. A) A subject without any detectable WMH. B) High signal intensity visible only within the posterior periventricular crossroads regions. C) Extensive high signal intensity noted within crossroads regions and surrounding white matter. Corresponding images D, E, and F, respectively demonstrate semi-automated segmentation results for WMH regions (in red).

Cerebral tissues were segmented automatically using in-house developed software specifically designed for preterm infants [16]. Regional structures were segmented manually by a single experienced rater, masked to clinical factors, using defined landmarks distinguished by spatial anatomy, shape, and image intensity. We have previously reported our detailed methods and reported high automated tissue segmentation accuracy and high manual segmentation intra- and inter-rater reliability (ICC range: 0.942 to 0.998) [16]. Briefly, Analyze 8.1 (Biomedical Imaging Resource, Mayo Clinic, Rochester, MN) was used for manual segmentation of the caudate/accumbens, lenticular nuclei, thalamus, hippocampi, amygdalae, corpus callosum, brain stem and cerebellum. Cortical gray matter (GM), cerebral white matter (WM), and cerebrospinal fluid (CSF) tissue classes were automatically segmented after correction for signal inhomogeneity. As we have described previously, this software systematically mislabeled regions of periventricular and subcortical white matter hyperintensities (WMH) as CSF, when WM intensity approached CSF intensity values. We manually relabeled these as WMH [16]. These regions include both normal periventricular fiber crossroads as well as pathologic DEHSI, which extend beyond these crossroads into surrounding periventricular and subcortical regions (Figure 1). The total brain tissue volume was defined as the combined volume of total GM and WM. For a given tissue/structure, the absolute volume was calculated as the total number of voxels containing the segmented area, multiplied by the planar dimensions of the voxels and the thickness of the brain slice. All volumes are presented as mL (equivalent to cm^3^).

### Main Outcome Measures

Our main outcome measures were volumes of total brain tissue and WMH. Secondary outcome measures included volumes of: 1) Cerebral (subcortical/periventricular) WM; 2) Cortical GM; 3) Total CSF (ventricular and extra-axial); 4) Cerebellum; 5) Corpus callosum; 6) Hippocampi; 7) Caudate/accumbens; 8) Lenticular nuclei; 9) Thalamus; 10) Amygdalae; 11) Brain stem; and 12) Total cranial volume (TCV - all brain tissues, including CSF).

### Statistical Analyses

For normally distributed regional volumes, we determined adjusted means and group differences between preterm and term controls using one-way ANOVA and linear regression, respectively. Regional volumes with a non-Gaussian distribution were analyzed after log transformation or using robust regression depending on the type of non-Gaussian distribution. Analyses controlling for PMA at MRI were conducted to investigate the magnitude of regional volumes differences between preterm infants and term infants at the same PMA at MRI scan. We also conducted analyses controlling for TCV in addition to PMA at MRI, to address whether the growth of specific regions of the brain is differentially affected in preterm newborns as compared to term controls when total head size and age at MRI are held constant. Last, because abnormal CSF volumes in ELBW infants can significantly skew head and total brain size, we also conducted analyses adjusted for total brain tissue volume (and PMA at MRI). All potential confounding variables were centered on their group means.

Based on prior evidence and biologic plausibility, 44 clinical factors were evaluated (see Table S1) for the preterm only antecedent analyses. A systematic manual variable selection approach was utilized to identify independent factors associated with regional brain volumes in the following four sequential time periods: variables present before or during pregnancy (antenatal), those present at birth (intrapartum), those measured on the first postnatal day (at birth), and factors occurring between the second postnatal day and discharge MRI exam (NICU). For each successive time interval, all factors with a *p*<0.25 in univariate analyses were tested together and retained if *p*<0.10 for further testing in the next period. If all factors did not reach this level of significance, then the variable with the lowest *p* value was retained and the process was repeated by testing the remaining factors together until none of the variables had a *p* value of <0.10. All models were first adjusted for PMA at MRI only to evaluate significant associations between antecedents and brain growth/development. To evaluate associations between clinical factors and global or regional brain injury/atrophy, it is important to control for differences in head size. Conducting analyses adjusting for TCV corrected for head size differences. Because TCV approximates head growth more closely than total brain tissue volume, adjusting with TCV would be sufficient if not for the residual hydrocephalus despite treatment observed in some preterm infants. We therefore conducted separate analyses adjusting for total brain tissue volume to overcome this shortcoming. Four dependent variables required log or square root transformations and an additional four with outliers were assessed using robust regression. Regression diagnostics were performed to verify that assumptions of regression testing were not violated.

A comparison of observed parameter estimates with distributions derived from a bootstrap procedure involving 10,000 resamples was performed to assess validity of the final model coefficients. For the 14 final models we developed, the variable estimates were within 0.3% to 10.3% of the mean of the bootstrap estimates, confirming internal validity. Two-sided *p* values of <0.01 were considered to indicate statistical significance. We performed all analyses using STATA 11.0 (Stata Corp., College Station, TX).

## Results

Of the 134 eligible ELBW infants, 11 were excluded because of poor quality MRI (motion artifacts or thicker slices) and one due to missed MRI exam. The remaining 122 infants were enrolled in this study. Excluded infants were similar to study ELBW infants in maternal characteristics and sex but were smaller on average –25.7 weeks gestational age and 672 g birth weight – and fewer infants were SGA (8%). Table 1 provides maternal and neonatal characteristics of the ELBW and term cohorts. ELBW survivors experienced high rates of complications and morbidities.

**Table 1:** Characteristics of the preterm and term cohorts.

Characteristics	Preterm (n = 122)	Term (n = 16)
Maternal hypertension, n (%)	43 (35)	1 (6)
Maternal chorioamnionitis, n (%)	39 (32)	0
Maternal antibiotics, n (%)	70 (57)	7 (44)
Antenatal steroids (any), n (%)	86 (70)	0
Gestational age, weeks	26.7±1.9	38.8±1.0
Birth weight, grams	771±144	3183±369
Males, n (%)	53 (43)	7 (44)
5-min Apgar score <5, n (%)	14 (11)	0
Small for gestation <10th%, n (%)	30 (25)	0
Outborn birth, n (%)	16 (13)	0
Pneumothorax, n (%)	6 (5)	0
Pulmonary hemorrhage, n (%)	3 (2)	0
White matter injury on cranial US, n (%)	13 (11)	0
Germinal matrix/IVH on cranial US, n (%)	22 (18)	0
Culture positive bacteremia, n (%)	51 (42)	0
Necrotizing enterocolitis, n (%)	8 (7)	0
Anticonvulsant therapy for seizures, n (%)	6 (5)	0
PDA surgery, n (%)	41 (33)	0
Postnatal corticosteroids for BPD, n (%)	22 (18)	0
Caffeine for apnea of prematurity, n (%)	108 (89)	0
Parenteral nutrition duration, days	38.3±26.7	0
Severe ROP (III or plus), n (%)	35 (29)	0
Surgical therapy for ROP, n (%)	29 (24)	0
Gastrointestinal surgery, n (%)	10 (8)	0
Positive pressure use at 36 weeks PMA, n (%)	46 (38)	0
Severe BPD, n (%)	63 (52)	0
Postmenstrual age at MRI, weeks	38.5±2.2	39.0±1.0

Plus-minus values are mean±SD.

As compared to healthy term infants, ELBW infants exhibited significantly decreased regional tissue and structural volumes in a majority of analyzed regions in unadjusted and adjusted analyses (Table 2). The mean total brain tissue volume for ELBW infants was 42.79 mL smaller (*p*<0.001) in analyses adjusted only for PMA at MRI and 24.29 mL smaller after additionally controlling for TCV. All measured regional WM and GM structures were also significantly smaller in ELBW infants contributing to this overall decrease in total tissue volume. These adverse changes were accompanied by a marked increase in mean CSF volume and a trend towards smaller TCV. Secondary adjustment with total brain tissue volume resulted in systematically smaller group differences in most regions with loss of statistical significance for several regions and larger adjusted cortical GM volume in preterm infants. ELBW infants were imaged using higher resolution sequence parameters but lower magnet strength as compared to their term counterparts. These opposing attributes contributed to comparable image quality and segmentation times.

**Table 2:** Regional Tissue and Structural Brain Volumes (mL) for the Preterm and Term Cohorts.

Region	Preterm Unadjusted Mean Volume(N = 122)	Term UnadjustedMean Volume(N = 16)	Group Difference in Means Adjusted forPMA at MRI (95% CI)	Relative Percent Difference	Group Difference in Means Adjusted for PMA at MRI & TCV(95% CI)	Relative PercentDifference	Group Difference in Means Adjusted for PMA at MRI & Total Brain Tissue (95% CI)	Relative Percent Difference
Total brain tissue	270.28±41.50	316.82±35.52	−42.79 (−62.85, −22.73)**	−14.3%**	−24.29 (−31.68, −16.90)**	−8.1%**	NA	NA
Cerebral white matter	124.06±18.80	146.61±16.44	−21.69 (−31.33, −12.04)**	−15.5%**	−13.75 (−18.85, −8.66)**	−9.9%**	−2.45 (−6.10, 1.20)	−1.9%
WMH ratioa,b	0.01 (0.00, 0.03)	0.01 (0.00, 0.02)	0.00 (−0.01, 0.01)	−3.6%	0.00 (−0.01, 0.01)	−17.4%	0.00 (−0.01, 0.01)	−0.1%
Cortical gray matter	105.11±19.12	119.79±16.08	−12.32 (−20.87, −3.77)*	−10.5%*	−5.41 (−9.33, −1.48)*	−4.8%*	4.54 (1.06, 8.02)	4.4%
Cerebro-spinal fluid^a^	78.91 (67.56, 94.49)	70.37 (55.82, 79.09)	18.11 (3.98, 34.65)**	27.8%**	20.61 (12.59, 29.52)**	34.4%**	25.36 (12.09, 40.90)**	43.2%**
Cerebellum	16.06±4.23	19.63±2.51	−3.18 (−5.16, −1.20)*	−17.3%*	−1.59 (−2.81, −0.37)	−8.7%	−0.014 (−1.41, 1.38)	−0.1%
Corpus callosum	1.02±0.30	1.55±0.35	−0.54 (−0.70, −0.38)**	−34.8%**	−0.43 (−0.57, −0.28)**	−29.4%**	−0.35 (−0.49, −0.21)**	−25.1%**
Hippocampi	1.34±0.25	1.52±0.25	−0.18 (−0.31, −0.05)*	−11.7%*	−0.11 (−0.23, 0.001)	−7.7%	−0.014 (−0.13, 0.10)	−1.1%
Caudate/accumbens^a^	3.15 (2.86, 3.59)	3.91 (3.57, 4.51)	−0.84 (−1.14, −0.53)**	−21.3%**	−0.58 (−0.79, −0.36)**	−15.3%**	−0.28 (−0.48, 0.06)	−8.0%
Thalamus	7.70±1.26	9.12±1.22	−1.37 (−2.03, −0.72)**	−15.1%**	−0.89 (−1.31, −0.47)**	−10.2%**	−0.18 (−0.54, 0.18)	−2.2%
Lenticular nuclei	5.43±0.85	6.69±0.75	−1.25 (−1.69, −0.80)**	−18.7%**	−0.96 (−1.29, −0.62)**	−15.2%**	−0.54 (−0.85, −0.22)**	−8.9%**
Amygdalae^a^	0.55 (0.45, 0.66)	0.66 (0.61, 0.74)	−0.13 (−0.20, −0.04)*	−18.5%*	−0.09 (−0.15, −0.01)	−13.2%	−0.03 (−0.09, 0.05)	−4.6%
Brain stem	5.77±0.82	7.18±0.66	−1.36 (−1.76, −0.95)**	−19.0%**	−1.08 (−1.35, −0.81)**	−15.6%**	−0.65 (−0.90, −0.40)**	−10.0%**
Total cranial volume	357.27±59.58	387.08±39.50	−23.62 (−51.07, 3.83)	−6.2%	N/A	N/A	N/A	N/A

Plus-minus values are mean±SD.

* *p*<0.01;

** *p*<0.001.

a Median (IQR).

b Reported as a ratio of WMH to cerebral white matter volume.

We detected and quantified WMH in 84% of ELBW and in 94% of term infants. There was no significant difference in the ratio of WMH to cerebral WM volume between the two groups (Table 2). However, in post hoc analyses, extensive WMH, defined as a WMH volume greater than 95^th^ percentile for term newborns (5.65 mL), what might be referred to as DEHSI, was observed in 15 ELBW infants as compared to one term control. The distribution of WMH in term infants was primarily observed in the frontal and occipital periventricular crossroads regions.

**Figure 2:**
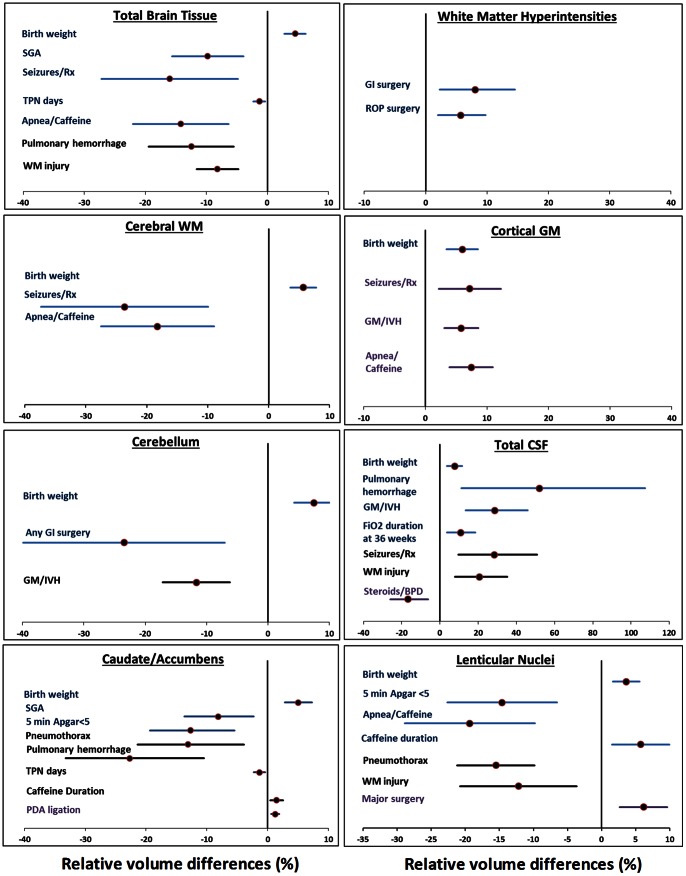
Relative mean volume differences (%) for antecedents with significant associations (*P*<0.01) in eight cerebral regions. Clinical factors that were significantly associated with cerebral regional volumes in each of eight regions from multivariable models are presented with relative volume differences (%) and 95% confidence intervals. Unique antecedents that were adjusted only for PMA at MRI are presented with blue error bars; additional antecedents from models adjusted for PMA at MRI and total cranial volume are in black bars and models adjusted for PMA at MRI and total brain tissue volume are presented with purple error bars.

In analyses controlling only for differences in PMA at MRI scan, four risk factors - SGA, seizures/anticonvulsant therapy, apnea/caffeine therapy, and duration of total parenteral nutrition - were significantly associated with smaller total brain tissue volume (relative difference: −1.4% to −15.0%). *p*<0.01; Figure 2; Table 3). Birth weight was directly associated with total brain tissue volume (4.5% per 100 g increase; *p*<0.001). Controlling the analyses for TCV identified two different risk factors for total brain tissue volume - pulmonary hemorrhage and WM injury on cranial US (*p*<0.01). The aforementioned five antecedents from the PMA at MRI only adjusted model were no longer significant when head size was controlled. Surgical therapy for ROP and gastrointestinal surgery for NEC and/or spontaneous intestinal perforation were significantly associated with increased WMH/cerebral WM volume ratio in all three adjusted analyses (Figure 2; Table 3).

**Table 3:** Clinical factors significantly associated with total brain tissue volume (mL) and WMH in multivariable regression models.

Brain Region	Antecedent	Model F statistic/Adjusted R^2^	Coefficient (95% CI) –Adjusted by PMA at MRI	Model F statistic/Adjusted R^2^	Coefficient (95% CI) – Adjusted by TCVand PMA at MRI	Model F statistic/Adjusted R^2^	Coefficient (95% CI) – Adjusted by Total Brain Tissue Volume and PMA at MRI
Total brain tissue	Birth weight	18.87**/0.47	10.65 (6.50, 14.80)**	108.09**/0.86	NS	NA	NA
	SGA status		−22.99 (−36.72, −9.26)*		NS		NA
	Duration parenteralnutrition (per 10d)		−3.12 (−5.43, −0.80)*		NS		NA
	Apnea/Caffeine therapy		−33.35 (−51.75, −14.95)**		NS		NA
	Seizures/Anticon-vulsant therapy		−37.59 (−63.93, −11.25)*		−17.84 (−31.60, −4.08)†		NA
	WM injury on cranial US		NS		−22.72 (−33.31, −13.13)**		NA
	Pulmonary hemorrhage		NS		−37.78 (−57.18, −18.38)**		NA
WMH^a^	GI surgery	5.24**/NV	8.08 (2.28, 14.56)*	5.37*/NV	7.57 (1.89, 13.89)*	4.17*/NV	8.25 (2.35, 14.86)*
	Severe ROP surgery		5.73 (2.01, 9.75)*		6.34 (2.51, 10.48)*		6.04 (2.14, 10.28)*

† *p* = 0.01 *p<0.01 **p<0.001.

NS – not significant; NA – not applicable; NV – no value (because utilized robust regression).

a Reported as a percentage mean difference in WMH to cerebral white matter volume (95% CI).

**Figure 3:**
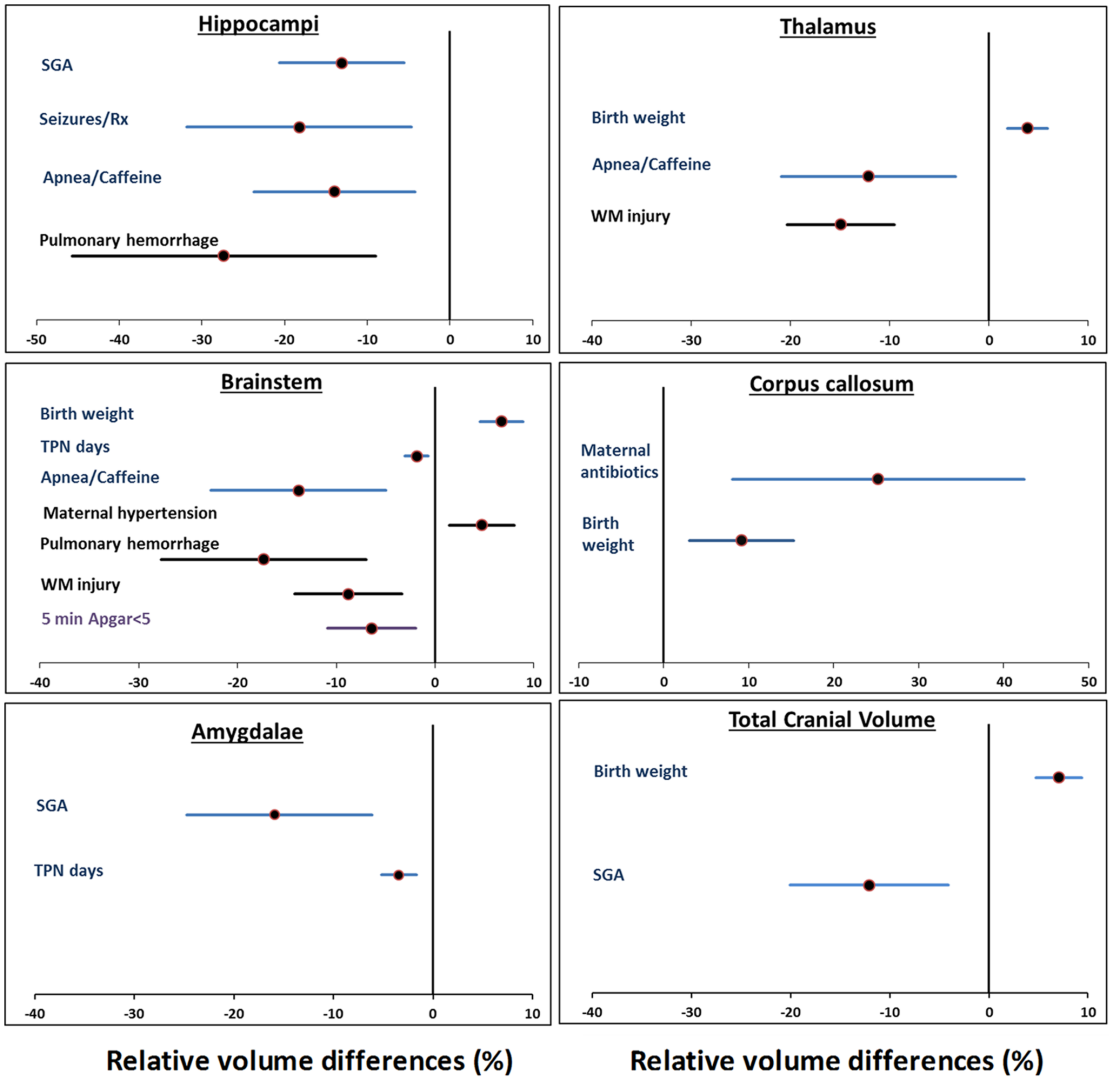
Relative mean volume differences (%) for antecedents with significant associations (*P*<0.01) in six additional cerebral regions. Clinical factors that were significantly associated with cerebral regional volumes in each of six regions from multivariable models are presented with relative volume differences (%) and 95% confidence intervals. Unique antecedents that were adjusted only for PMA at MRI are presented with blue error bars; additional antecedents from models adjusted for PMA at MRI and total cranial volume are in black bars and models adjusted for PMA at MRI and total brain tissue volume are presented with purple error bars.

In developing multivariable models for regional brain volumes, we identified significant associations with 10 clinical risk factors, 8 of which were from the NICU period (Table S2; Figure 2; Figure 3). In all, such clinical factors accounted for approximately half of the variance in regional brain volumes (highest adjusted R^2^∶0.49). Of these, pulmonary hemorrhage, WM injury on cranial US, treated seizures, apnea/caffeine therapy, and duration of TPN exhibited the strongest associations and widespread detrimental effects on regional volumes. Duration of caffeine therapy however, was associated with larger caudate/accumbens and lenticular nuclei volume. Birth weight was positively correlated with a large majority of the regional volumes while SGA was negatively associated with volumes of several nuclei and total brain tissue (Figure 2; Figure 3 and Table S2). Chorioamnionitis, antenatal steroids, sex, gestational age (GA), and low dose postnatal steroids were not significantly associated with regional volumes.

## Discussion

We observed globally smaller cerebral tissue and structural volumes in a cohort of high-risk ELBW infants as compared to term controls. These marked reductions were accompanied by a compensatory increase in CSF volume and smaller total brain size, suggesting cerebral atrophy was partially responsible for smaller volumes. Cortical and deep nuclear GM volume decrements were less prominent than cerebral WM volume deficits. Controlling for head or brain size resulted in smaller group differences. We quantified WMH and observed comparable adjusted volumes between groups. Though not reported previously, presence of high signal intensity regions in healthy term controls – primarily in the anterior and posterior periventricular WM regions – likely represents normal developmental crossroads. The developmental abundance of hydrophilic extracellular matrix and multiple crossing fibers in these regions contributes to this bright signal on T2 weighted MRI scans [14]. Kidokoro et al. [15] reported a 12% incidence of invisible crossroads in their large cohort resulting from extensive high signal intensity in very preterm infants; this finding correlated significantly with mental development scores at 2 years corrected age. We observed a similar incidence of extensive WMH, which is likely pathologic (i.e. DEHSI), in our ELBW infants. Our findings of tissue volume abnormalities are consistent with recent observations [5]–[7], [23], [27]; additional measurements of WMH and deep nuclear GM volumes extend our understanding of brain development following preterm birth and neonatal intensive care.

Brain volume abnormalities and DEHSI are the most frequent findings in ELBW infants on term-equivalent MRI scans [5]–[8]. We examined a diverse profile of well-defined antecedents and correlated them with regional brain and WMH volumes in ELBW infants. Birth weight was positively associated with total and regional brain volumes. While WM injury and SGA status have been previously linked with total or regional tissue volumes [5], [7], [28], treated seizures, apnea/caffeine therapy, TPN duration, and pulmonary hemorrhage have not. Apnea of prematurity treated with caffeine therapy was associated with smaller total and regional tissue volumes. Duration of caffeine therapy however, was associated with larger caudate/accumbens and lenticular nuclei volume, suggesting the detrimental association with apnea/caffeine therapy may have been a result of the underlying condition. Analyses adjusted for head size (TCV) would be expected to identify risk factors more closely associated with brain injury and secondary atrophy rather than delayed brain growth. If risk factors resulted in acute brain injury (e.g. pulmonary hemorrhage), they would be expected to secondarily reduce brain tissue size over time with comparably less effect on total head size (due to compensatory CSF increase). Such risk factors would be masked unless head size differences were controlled.

Intrauterine growth retardation has been the only significant antecedent of WMH reported to date [12]. In our cohort, surgery for ROP and surgery for NEC or spontaneous intestinal perforation were associated with a relative mean increase in the ratio of WMH/cerebral WM by 5.7% and 8.1%, respectively. Information about operative and post-operative medications or complications unfortunately was not collected to further elucidate the role of such factors in the pathogenesis of surgery-associated brain injury. These risk factors and neonatal surgery/anesthesia in general have been adversely associated with NDI in multiple studies [29]–[33]. Their association with WMH adds more evidence that when it is severe/diffuse, it may represent a non-cystic form of diffuse white matter damage [34]. Presence of a small volume of WMH, primarily in the crossroads regions, is likely normal. Most of the term infants exhibited small amount of WMH in such crossroads regions only. The 15 ELBW infants with volumes above the 95^th^ percentile for term controls may be at the highest risk for NDI. Further improvements in WMH segmentation may help resolve this important debate.

The limitations of our study include a single center population and low incidences of a few antecedents. Head size corrected term and preterm brain volumes differences following TCV adjustment may have been exaggerated because some infants likely exhibited residual hydrocephalus despite therapeutic interventions. Adjustment with total brain tissue volume to address this concern could have alternatively reduced group differences because differential regional growth or volume deficits in several smaller regions would reduce total brain tissue volume. Therefore, the true head size corrected group differences are likely in between these two measures. The large reductions we observed were likely secondary to the high degree of illness of our cohort as compared to previous cohorts [7], [35], where brain volume was generally conserved in infants without neonatal morbidities. For the preterm analyses, it remains unclear how best to adjust for individual differences in head size in preterm infants. As such, we performed adjustments with both TCV and total brain tissue volume, resulting in discrepancies in significant regional antecedents. A longitudinal cohort study with serial brain volume measurements is needed to resolve these differences. An additional limitation relates to our use of different magnetic field strengths for the preterm and term groups. Imaging at different field strengths results in regionally variant geometric distortions with resulting volumetric differences [36], [37]. However, such differences are likely smaller as compared to the known biologic structural differences between very preterm and healthy full-term newborns imaged at term. For example, the percent tissue volume differences we report (adjusted only for PMA at MRI) fall between those reported by Inder et al. [5] and Thompson et al. [7]. Moreover, the effect of this potential confounder should have been further minimized with our adjusted analyses, controlling for TCV and total brain tissue volume. This limitation did not affect the preterm only risk factor analyses. Similar to other observational cohorts, our study is prone to bias such as confounding by indication and residual confounding that can only be resolved with a randomized trial design. Additionally, despite using a significance threshold of <0.01 and confirming internal validity, multiple comparisons may have contributed to some false positives, such as the associations between PDA ligation or major surgery and larger GM nuclei. Additionally, only about a third of the perinatal factors that were significant at a p<0.01 remained significant at a p<0.001 level.

The strengths of our study include, evaluation of a relatively large unselected cohort of high-risk ELBW infants, comparison with term controls, quantification of WMH and deep nuclear GM volumes, examination of a diverse group of risk factors, and internal validation with bootstrapping. Our age corrected global and structural measurements for term controls closely match post-mortem volume measurements reported by Nisari et al. [38] and in vivo MRI measurements by Clouchoux and colleagues [39] but are smaller than those reported by Thompson et al. [7] in neonates. These discrepancies may relate to study differences in subject race, image acquisition, or post-processing methods utilized.

We identified multiple clinical factors that were associated with one or more regional volumes. These findings highlight the utility of brain volume measurements and the multifactorial nature of brain injury/development in ELBW infants [34]. Additional follow-up studies demonstrating robust correlations between regional volumes and NDI are required to confirm the value of regional volumes as surrogate endpoints for NDI [40]. Such measures are of particular importance to the ELBW population where outcomes cannot otherwise be accurately ascertained until at least 2 years corrected age for motor disabilities and school age for cognitive and behavioral impairments [41], [42].

### Conclusions

Extremely preterm infants exhibit widespread regional brain volume abnormalities that are sensitive to multiple perinatal risk factors and neonatal morbidities/interventions. Our findings likely reflect the multifactorial sequelae of poor antenatal/postnatal nutrient delivery, postnatal morbidities, and adverse treatment effects on brain development. Brain growth measurements in ELBW infants can advance our understanding of perinatal brain injury and development and illuminate potential avenues to prevent cerebral macrostructural abnormalities.

### Data Accessibility Statement

Any researcher wishing to access our data may do so by stating their research objective(s) and desire to access the data in an email to the corresponding author.

## Supporting Information

Table S1 **Infant and maternal antecedent factors evaluated in multivariable regression models.**
(DOCX)

Table S2 **Clinical factors significantly associated with brain regional volumes (mL) in multivariable regression models.**
(PDF)
