# Supplementary material for: Perinatal Factors and Regional Brain Volume Abnormalities at Term in a Cohort of Extremely Low Birth Weight Infants
Source: PLoS One. 2013 May 9;8(5):e62804. doi: 10.1371/journal.pone.0062804 (PMC3650008; doi:10.1371/journal.pone.0062804)
Supplement: Table S1 — Infant and maternal antecedent factors evaluated in multivariable regression models. (DOCX) [file pone.0062804.s001.docx]

Table S1: Infant and maternal antecedent factors evaluated in multivariable regression models.

Variable	Definition
**Antepartum**	
Maternal age	Maternal age at delivery
Maternal insurance	Public or no-insurance status upon admission to NICU
Maternal hypertension	Chronic or pregnancy induced hypertension recorded in mother's chart
Maternal insulin-dependent diabetes	Diabetes mellitus requiring insulin for control, diagnosed during or prior to present pregnancy.
Antepartum hemorrhage	Placenta previa, abruption or threatened abortion resulting in bleeding, which can be external (vaginal bleeding) or occult (retroplacental clot), other than bloody show, after 20 weeks of pregnancy.
Sex	Male, female, or ambiguous sex of the infant
Multiple birth	Multiple gestation pregnancy (twins or greater, live or stillborn)
**Intrapartum**	
Chorioamnionitis	Clinical or histologic chorioamnionitis documented in mother's medical records.
Antenatal steroids	Exposure to one or more antenatal doses of antenatal steroids to accelerate maturity.
Maternal antibiotics	Use of any maternal antibiotics during the admission resulting in this delivery.
**At Birth**	
Out born birth	Born outside Children's Memorial Hermann Hospital
Gestational age (GA)	Gestational age in completed weeks and days by best obstetric estimate in the following hierarchy – early prenatal ultrasound, last menstrual period, second trimester ultrasound; the best neonatologist estimate if best obstetric estimate was unavailable.
Birth weight (BW)	Birth weight in grams from labor and delivery or nursery admission records
Small for gestational age (SGA)	Gestational age below the 10th percentile for birth weight as defined by Kramer et al. (Pediatrics. 2001; 108:35)
Resuscitation/stabilization in delivery room requiring intubation	Tracheal intubation in delivery room to provide positive pressure ventilation.

Resuscitation/stabilization in delivery room requiring chest compressions	External pressure over central chest to contract heart in delivery room.
**Variable**	**Definition**
Apgar score at 5 minutes <5	Five minute Apgar score of <5
Oxygen/positive pressure support required for >6 hrs. within first 24 hours	Infant required supplemental oxygen (FiO2 > 0.21) and/or positive pressure support continuously for more than 6 hours within the first 24 hours.
**NICU**	
Pneumothorax	Documented pneumothorax or collection of air in the pleural space with displacement of the lung away from the chest wall.
Pulmonary hemorrhage	Bright red blood per endotracheal tube associated with clinical deterioration.
Postnatal corticosteroids for BPD	Any doses or course of systemic steroids to prevent or treat bronchopulmonary dysplasia. Does not include steroids for extubation and/or stridor, or inhaled steroids.
Patent ductus arteriosus (PDA)	Echocardiographic evidence of PDA with documentation of left to right ductal shunting or clinical evidence by continuous murmur, hyperdynamic precordium, bounding pulses, wide pulse pressure, congestive heart failure, chest X-ray changes, and/or increase oxygen requirement.
Indomethacin for PDA	Receipt of one or more doses of treatment indomethacin to close a diagnosed PDA (does not include prophylactic indomethacin, a therapy that was not prescribed in our NICU during the study period).
PDA ligation	Surgical ligation to close the PDA
**Treated seizures**	Seizures (clinical and/or electrographic) treated with anticonvulsant for more than 72 hours.
Germinal matrix/ intraventricular hemorrhage (IVH) on cranial ultrasound	Blood/echo-density in germinal matrix/sub-ependymal area or intraventricular without ventricular dilation on cranial ultrasound (US) within 28 days of life
White matter injury on cranial US within 28 days of birth	Any of the following: blood/echo-density in the parenchyma; ventricular dilation with or without blood; cystic areas/echo-lucencies in the parenchyma consistent with periventricular leukomalacia or porencephalic cysts within 28 days of birth
Culture positive sepsis/bacteremia	Positive culture of blood concurrent with clinical signs of septicemia
Postnatal sepsis	Culture positive sepsis or culture negative clinical infection treated with antibiotics for ≥5 days

Days to first enteral feedings	Number of days before enteral feedings were initiated the first time
Total days of total parenteral nutrition support	Number of days in which the infant received parenteral alimentation including amino acids or lipid solution.
Necrotizing enterocolitis	Proven Bell Stage II or III necrotizing enterocolitis (NEC)
Necrotizing enterocolitis requiring surgery	Bell Stage IIIB NEC requiring surgery
**Variable**	**Definition**
Any gastrointestinal (GI) surgery	Spontaneous gastrointestinal perforation or NEC requiring surgery
Major surgery	Gastrointestinal surgery for NEC or spontaneous perforation, PDA ligation surgery, or any other major surgery requiring anesthesia and performed prior to brain MRI (e.g. fundoplication, ventricular shunt).
Retinopathy of prematurity (ROP) stage 3 or plus disease	ROP stage 3 or worse or plus disease (enlargement of the posterior veins of the retina and tortuous arterioles) noted in either eye.
Surgery for retinopathy of prematurity	Retinal ablation, scleral buckle, vitrectomy or other surgery in either eye for treatment of ROP
Caffeine therapy	Any use of caffeine for treatment of apnea of prematurity, for ventilator dependence, and/or for prevention of extubation failure
Duration of caffeine	Duration of caffeine use prior to brain MRI
Severe bronchopulmonary dysplasia	Severe bronchopulmonary dysplasia as defined by NIH consensus definition; oxygen need for ≥28 days plus ≥30% effective oxygen supplementation and/or positive pressure at 36 weeks PMA
Positive pressure use at 36 weeks PMA	Receiving mechanical ventilation or nasal continuous positive airway pressure at 36 weeks PMA (nasal SIMV was not used at our institution during study period)
Days of mechanical ventilation up to 36 weeks PMA	Number of days of conventional or high frequency mechanical ventilation administration prior to and including 36 weeks PMA
Days of positive pressure up to 36 weeks PMA	Number of days of conventional or high frequency mechanical ventilation and nasal continuous positive airway pressure administration prior to and including 36 weeks PMA
Duration of supplemental oxygen up to 36 weeks PMA	Number of days of supplemental oxygen prior to and including 36 weeks PMA
