# Supplementary material for: Perinatal Factors and Regional Brain Volume Abnormalities at Term in a Cohort of Extremely Low Birth Weight Infants
Source: PLoS One. 2013 May 9;8(5):e62804. doi: 10.1371/journal.pone.0062804 (PMC3650008; doi:10.1371/journal.pone.0062804)
Supplement: Table S2 — Clinical factors significantly associated with brain regional volumes (mL) in multivariable regression models. (PDF) [file pone.0062804.s002.pdf]

Table S2. Clinical factors significantly associated with brain regional volumes (mL) in multivariable regression models.

Antecedent	Region	Mean Volume Difference (95% CI) Adjusted by PMA at MRI	Mean Volume Difference (95% CI) Adjusted by PMA at MRI and TCV	Mean Volume Difference (95% CI) Adjusted by PMA at MRI and Total Brain Tissue Volume
Antenatal				
**Maternal hypertension**	Brainstem		0.25 (0.08, 0.43)*	
Intrapartum				
**Maternal antibiotic therapy**	Corpus callosum	0.15 (0.05, 0.25)*	0.15 (0.05, 0.24)*	0.16 (0.06, 0.25)*
At Birth				
**Apgar score <5 at 5 min.**	Lenticular nuclei	-0.69 (-1.08,-0.31)**	-0.57 (-0.88, -0.26)**	-0.46 (-0.5, -0.18)*
	Caudate/accumbens	-0.07 (-0.11, -0.03)*		-0.14 (-0.21, -0.06)*
	Brainstem			-0.36 (-0.61, -0.11)*
**Birth weight (per 100g increase)**	Total brain tissue	10.65 (6.50,14.80)**		
	Cortical GM	4.47 (2.58, 6.36)**		
	Cerebral WM	5.52 (3.46, 7.58)**		
	Total CSF	0.57 (0.28, 0.86)**		
	Cerebellum	0.88 (0.50, 1.25)**		
	Caudate/accumbens	0.03 (0.02, 0.04)**		
	Corpus callosum	0.05 (0.02, 0.09)*		
	Thalamus	0.27 (0.13, 0.41)**		
	Lenticular nuclei	0.17 (0.08, 0.26)**		
	Brainstem	0.27 (0.18, 0.36)**		
	Total cranial volume	17.66 (11.83, 23.49)**		
**Small for gestational age (<10th%)**	Total brain tissue	-22.99 (-36.72, -9.26)*		
	Hippocampus	-0.18 (-0.28, -0.07)*		
	Caudate/accumbens	-0.01, (-0.02, -0.002)*		

	Amygdalae	-0.07 (-0.10, -0.03)*		
	Total cranial volume	-30.24 (-50.23, -10.25)*		
NICU				
**Pneumothorax**	Lenticular nuclei		-0.71 (-1.21, -0.21)*	-0.76 (-1.21, -0.30)*
	Caudate/accumbens		-0.18 (-0.29, -0.05)*	
**Pulmonary hemorrhage**	Total brain tissue		-34.70 (-54.10, -15.31)**	
	Cerebral WM		-16.05 (-27.63, -4.47)*	
	Total CSF	4.01 (0.87, 8.29)*		
	Caudate/accumbens		-0.31 (-0.45, -0.14)*	
	Hippocampi		-0.38 (-0.63, -0.12)*	
	Brainstem		-0.93 (-1.48, -0.37)*	
**WM injury on cranial US (within 28 days of birth)**	Total brain tissue		-22.72 (-32.19, -13.25)**	
	Total CSF		3.42 (1.27, 5.81)*	
	Thalamus		-1.27 (-1.73, -0.81)**	-0.69 (-1.05, -0.32)**
	Lenticular nuclei	-0.72 (-1.12, -0.32)*	-0.91 (-1.24, -0.57)**	-0.66 (-0.97, -0.36)*
	Brainstem		-0.47 (-0.76, -0.18)*	
**Germinal matrix/intra-ventricular hemorrhage**	Cortical GM			5.35 (2.80, 7.91)**
	Cerebellum		-2.14 (-3.15, -1.14)**	-2.11 (-3.23, -1.00)**
	Total CSF	2.21 (1.04, 3.54)**		4.01 (1.74, 6.57)**
**Treated seizures**	Total brain tissue	-37.59 (-63.93, -11.25)*		
	Cerebral WM	-22.97 (-36.28, -9.66)*	-15.65 (-25.03, -6.26)*	
	Cortical GM			6.60 (1.98, 11.22)*
	Total CSF		4.70 (1.58, 8.35)*	
	Hippocampus	-0.24 (-0.43, -0.06)*		
**Apnea/Caffeine therapy**	Total brain tissue	-33.35(-51.75, -14.95)*		
	Cerebral WM	-17.71 (-26.73, -8.68)*	-12.60 (-19.13, -6.07)*	
	Cortical GM			6.81 (3.55, 10.07)**

	Hippocampus	-0.19 (-0.32, -0.06)*		
	Thalamus	-0.84 (-1.45, -0.23)*		
	Lenticular nuclei	-0.92 (-1.37, -0.47)**		
	Brainstem	-0.55 (-0.91, -0.20)*		
**Caffeine therapy duration (per 30 days increase)**	Caudate/accumbens		0.06 (0.02, 0.10)*	0.07 (0.03, 0.11)*
	Lenticular nuclei	0.27 (0.07, 0.47)*		
**Parenteral nutrition duration (per 10 days increase)**	Total brain tissue	-3.12 (-5.43, -0.80)*		
	Caudate/accumbens		-0.02 (-0.03, -0.01)*	
	Amygdalae	-0.01 (-0.02, -0.007)*		
	Brainstem	-0.07 (-0.12, -0.03)*		
**Corticosteroids for BPD**	Total CSF			-2.67 (-4.16, -1.00)*
**PDA surgical ligation**	Caudate/accumbens			0.13 (0.06, 0.21)**
**Major surgery**	Lenticular nucleus			0.36 (0.16, 0.56)*
**GI surgery**	Cerebellum	-2.76 (-4.70, -0.83)*		
	WMH/Cerebral WM[a]	8.08 (2.28, 14.56)*	7.57 (1.89, 13.89)*	8.25 (2.35, 14.86)*
**Surgical therapy for Severe ROP**	WMH/Cerebral WM[a]	5.73 (2.01, 9.75)*	6.34 (2.51, 10.48)*	6.04 (2.14, 10.28)*
**Duration of oxygen supplementation at 36 weeks PMA (per 30 days)**	Total CSF	0.79 (0.27, 1.31)*		

*p <0.01 **p <0.001

[a] Reported as a percentage mean difference in WMH to cerebral white matter volume (95% CI).
